# Fabry disease and sleep disorders: a systematic review

**DOI:** 10.3389/fneur.2023.1217618

**Published:** 2023-10-05

**Authors:** Bartlomiej Blaszczyk, Mieszko Wieckiewicz, Mariusz Kusztal, Monika Michalek-Zrabkowska, Gabriella Lachowicz, Grzegorz Mazur, Helena Martynowicz

**Affiliations:** ^1^Student Research Club No K133, Faculty of Medicine, Wroclaw Medical University, Wrocław, Poland; ^2^Department of Experimental Dentistry, Wroclaw Medical University, Wrocław, Poland; ^3^Department of Nephrology and Transplantation Medicine, Wroclaw Medical University, Wrocław, Poland; ^4^Department of Internal Medicine, Occupational Diseases, Hypertension and Clinical Oncology, Wroclaw Medical University, Wrocław, Poland

**Keywords:** Fabry disease (FD), sleep disorders, excessive daytime sleepiness (EDS), obstructive sleep apnea (OSA), central sleep apnea (CSA)

## Abstract

**Background:**

Fabry disease (FD) is an X-chromosome-linked disorder characterized by a reduced or complete absence of the enzyme α-galactosidase, resulting in the accumulation of lysosomal globotriaosylceramide. Despite the presence of these deposits in multiple organs, the problem of sleep disorders within this population has very rarely been documented.

**Objective:**

This study aimed to investigate the types and prevalence of sleep disorders among patients with FD.

**Methods:**

Screening of the following medical databases using key terms was performed on 10 February 2023: PubMed, Scopus, and Embase. A total of 136 records were identified. The quality assessment of the studies was conducted by using tools from the National Institutes of Health (NIH) and critical appraisal tools from the Joanna Briggs Institute (JBI).

**Results:**

The study included nine studies on sleep disorders in patients with FD. The overall quality of the majority of these studies was assessed as either poor or fair. Among 330 patients, there was a slightly higher representation of female patients (56%). Sleep problems manifested 4–5 years after the onset of FD and sometimes even after 10–11 years. Genotypes of disease associated with sleep problems were rarely described. Within the FD population, the most commonly reported conditions were excessive daytime sleepiness (EDS) as well as obstructive and central sleep apnea (OSA, CSA). However, EDS occurred more frequently in FD patients, while the prevalence of OSA and CSA was within the ranges observed in the general population. The studies included indicated a lack of association between organ impairment by primary disease and EDS and OSA. The effectiveness of enzyme replacement therapy (ERT) in treating sleep disorders was not demonstrated.

**Conclusion:**

The findings of this report revealed the presence of many sleep-related disorders within the FD population. However, very few studies on this subject are available, and their limited results make it difficult to truly assess the real extent of the prevalence of sleep disturbances among these individuals. There is a need to conduct further studies on this topic, involving a larger group of patients. It is important to note that there are no guidelines available for the treatment of sleep disorders in patients with FD.

## 1. Introduction

Lysosomal storage diseases (LSDs) are congenital metabolic defects that impair lysosome function. LSDs are autosomal recessive disorders, several of which are linked to the X-chromosome. In general, mutations of lysosomal genes result in the accumulation of sphingolipids, mucopolysaccharides, or glycoproteins inside the lysosome, ultimately leading to cell damage and death. LSDs cover a group of 70 disorders, with Fabry disease (FD) being the most prevalent manifestation (1).

FD is an X-chromosome-linked disease that is marked by a reduced or complete absence of the α-galactosidase enzyme, resulting in the accumulation of lysosomal globotriaosylceramide (Gb3). In Europe, the disease affects between 1/3,100 and 1/117,000 individuals (2). However, these data may not reflect the actual prevalence as instances of the disease exist with partially active enzymes or within female patients carrying a defective gene (3). The disease manifests in different forms based on enzyme activity levels and the extent of mutation of the galactosidase alpha gene (GLA). The classic, severe clinical form is found only in men and manifests itself as early as childhood/teenage years (4). Atypical variants of the disease retain some residual α-galactosidase A activity, leading to these patients not exhibiting all of the described symptoms. The non-classic form affects both men and women and is characterized by a late clinical onset or even an asymptomatic course. This disease further distinguishes between cardiac and renal variations, involving specific organs only (5). It should be kept in mind that regardless of the variant, FD is a progressive disease with a reduced life expectancy. The median survival age for men is 50–55 years, while for women, it is 70 years (6).

Usually, the initial reason for seeking medical help is the presence of acute burning pain in the distal parts of the limbs, a condition referred to, in literature, as acroparesthesia. This neuropathic pain, however, may occur anywhere in the body. These pain episodes can be triggered by factors such as physical activity, ambient temperature, stress, or meals (7). In the classic presentation of the disease, characteristic reddish–purple skin lesions known as angiokeratoma are also observed, usually in the area around the umbilicus, trunk, and thighs. These lesions may spread to the facial area, impacting the appearance, which can be even more affected by occasional bleeding from the lesions (8). Tubulointerstitial kidney injury develops progressively, leading to renal failure and necessitating dialysis therapy (9). Cardiovascular symptoms are present in most patients who tend to develop left ventricular hypertrophy, myocardial fibrosis, and conduction abnormalities leading to arrhythmias (10). Neurological complications of the disease include memory impairment and headaches. The prevalence of vascular incidents including transient ischemic attacks (TIA), vascular dementia, and ischemic strokes is significantly higher in this group of patients (11).

Despite lysosomal deposits impacting many organs, the problem of sleep disorders in these patients has rarely been documented in studies on FD. Studies examining the quality of life in this patient group have reported daytime fatigue and sleep problems (12, 13). Furthermore, it is worth noting that no systematic reviews related to this clinical issue were found. Given these aspects, the aim of this systematic review was to investigate the type and prevalence of sleep disorders in patients with FD.

## 2. Methods

To write this systematic review, we conducted a literature search following the guidelines of the Preferred Reporting Items for Systematic Reviews and Meta-Analyses 2020 (PRISMA 2020) (14, 15). However, it is important to note that this systematic review was not registered.

### 2.1. Eligibility criteria

To be incorporated into this systematic review, studies needed to involve patients with FD, along with a description of their sleep disorders. Sleep disorders can be assessed by a wide range of medical methodologies, ranging from interviews or appropriate questionnaires to instrumental examinations. All age groups, genders, and FD variations were taken into consideration. Only original research was sought, and thus, reviews, book chapters, or comments were excluded. The following exclusion criteria as also applied: non-English records and the absence of sleep problems in FD patient groups.

### 2.2. Search strategy and study selection

Our search strategy was based on screening three medical databases, namely, PubMed, Scopus, and Embase (MEDLINE) by using key terms such as “Fabry disease” OR “Anderson–Fabry disease” AND “sleep” OR “insomnia” OR “sleeping problems” OR “restless leg syndrome” OR “snoring” OR “night.” On 10 February 2023, two authors (BB and HM) independently identified relevant records. Once studies were identified, duplicate studies were excluded from the review. To determine the content of each article, the same authors studied the titles and abstracts. By using this method, we excluded studies that were seen to be irrelevant for the purpose of our review. All identified reports were made available for retrieval. In the process of determining eligibility for inclusion in the final systematic review, the full texts of the remaining articles were read, and the results were compared by the authors. The third author (MW) was responsible for solving potential conflicts. As per the exclusion criteria outlined in Section 2.1, ultimately, only nine studies met the requirements for inclusion in this systematic review.

### 2.3. Data extraction and quality assessment

After the qualification of the final chosen studies for this review, two authors (BB and HM) extracted relevant data. The following information from the studies was taken into account: study authors, country of residence of patients diagnosed with FD, number of groups tested, patients' ages and genders, types of sleep disturbances, methods used to confirm the presence of sleep disorder, and FD variants. These data are shown in Table 1.

**Table 1:** General information of included studies demonstrating sleep disorders in Fabry disease patients.

**References**	**Country where the study was conducted**	**Patients' gender and number of cases**	**Patients' age**	**Patients' BMI**	**Enzyme replacement therapy receiving**	**Fabry disease variants**	**Sleep disturbances**	**Methods to confirm sleep problems**
Rosa Neto et al. (16)	Brazil	21 female and 16 male participants	42.1 ± 17.7 years old in female participants, 44.4 ± 11.6 years old in male participant	None	22 patients	Classic variants embraced: C142R, A156D, L180F, R227X, W262X, G271A, P293S, Y264SX mutations	17x insomnia/unrefreshing sleep, 22 × quality sleep disturbances	Medical interview, Pittsburgh, Sleep Quality Index
Gaisl et al. (17)	Switzerland	35 female and 17 male participants	42.1 ± 14.2 years old	23.3 ± 3.5 kg/m^2^	32 patients	Not reported	10 × obstructive sleep apnea, 7.9 ± 4.0 points for excessive daytime sleepiness	Respiratory polygraphy, Epworth Sleepiness Scale
Vallim et al. (18)	Brazil	10 female and 6 male participants	40.1 ± 12.9 years old	None	11 patients	Not reported	10 × poor sleep quality	Actigraphy, Pittsburgh Sleep Quality Index
Talbot et al. (19)	Australia	20 male participants	43.9 ± 10.7 years old	24.3 ± 3.8 kg/m^2^	16 patients	Phenotypes: 59% having cardiomyopathy and 37% cerebrovascular disease	8 × obstructive sleep apnea, 15 × restless legs syndrome, 19 × periodic limb movement in sleep, 7 × excessive daytime sleepiness	Polysomnography, Epworth Sleepiness Scale
Franzen et al. (20)	Switzerland	35 female and 17 male participants	42.8 ± 14.7 years old	23.4 ± 3.6 kg/m^2^	32 patients	Not reported	10 × obstructive sleep apnea, 3 × central sleep apnea, 7 × excessive daytime sleepiness	Respiratory polygraphy, Epworth Sleepiness Scale
Löhle et al. (21)	England	60 female and 50 male participants	49.0 ± 16.0 years old	None	80 patients	Not reported	29 × REM sleep behavior disorder, 28 × excessive daytime sleepiness	REM Sleep Behavior Disorder Screening Questionnaire, Epworth Sleepiness Scale
Duning et al. (22)	Germany	11 female and 12 male participants	48 ± 19 years old with sleep apnea, 46.0 ± 22.0 years old without sleep apnea	24.2 ± 8.2 kg/m^2^ with sleep apnea, 26.2 ± 9.8 kg/m^2^ without sleep apnea	23 patients	Not reported	5 × central sleep apnea with Cheyne–Stokes respiration, 2 × obstructive sleep apnea, 2 × above disorders together, 13.5 ± 8.1 points for excessive daytime sleepiness in CSA group, 9.4 ± 9.2 points for excessive daytime sleepiness without in group without CSA	Polysomnography, Medical interview, Epworth Sleepiness Scale
Rosa Neto et al. (23)	Brazil	11 female and 8 male participants	40.7 ± 15.1 years old	None	2 patients	Non-classic mutations: A143T and R118C.	9 × insomnia/unrefreshing sleep, 10 × quality sleep disturbances	Medical interview, Pittsburgh Sleep Quality Index
Duning et al. (24)	Germany	1 female participant	56 years old	None	1 patient	Not reported	1 × central sleep apnea with Cheyne–Stokes respiration, 1 × excessive daytime sleepiness	Polysomnography, Epworth Sleepiness Scale, Multiple sleep latency test

BMI, body mass index; REM, rapid eye movement; CSA, central sleep apnea.

The inclusion criteria encompassed a wide range of study types. Therefore, a number of tools were used to assess the quality of the studies. Tools established by the National Institutes of Health (NIH) were used to evaluate cohort and cross-sectional studies (25). Case–control studies were assessed using methodologies from the same institution. For cohort and cross-sectional research, a critical appraisal was done on the basis of 14 questions pertaining to study the conduct. Meanwhile, case–control studies were examined for bias on the basis of 12 different categories. Responses to each question could range from “yes,” “no,” “cannot determine,” “not applicable,” to “not reported.” The overall quality of the studies could be categorized as one of the following: “good” which indicates a low risk of bias, “poor” which equates to a high risk of bias, and “fair” which indicates a moderate risk of bias. Specific criteria for overall assessment were not formulated, considering each study's unique details requiring individual assessment. The assessment for bias in the case report was done according to the Joanna Briggs Institute (JBI) critical appraisal tool (16). Quality assessment was done with a designation of “high” assigned if the study received 7 or more “yes” answers out of the 8 designated criteria. “Moderate” quality was attributed to studies that received 5–6 “yes” responses, while quality was considered to be “low” when ≤ 4 positive answers were received. Two researchers (BB and HM) performed the above procedure and compared the final results during the discussion.

## 3. Results

### 3.1. Search results

**Figure 1:**
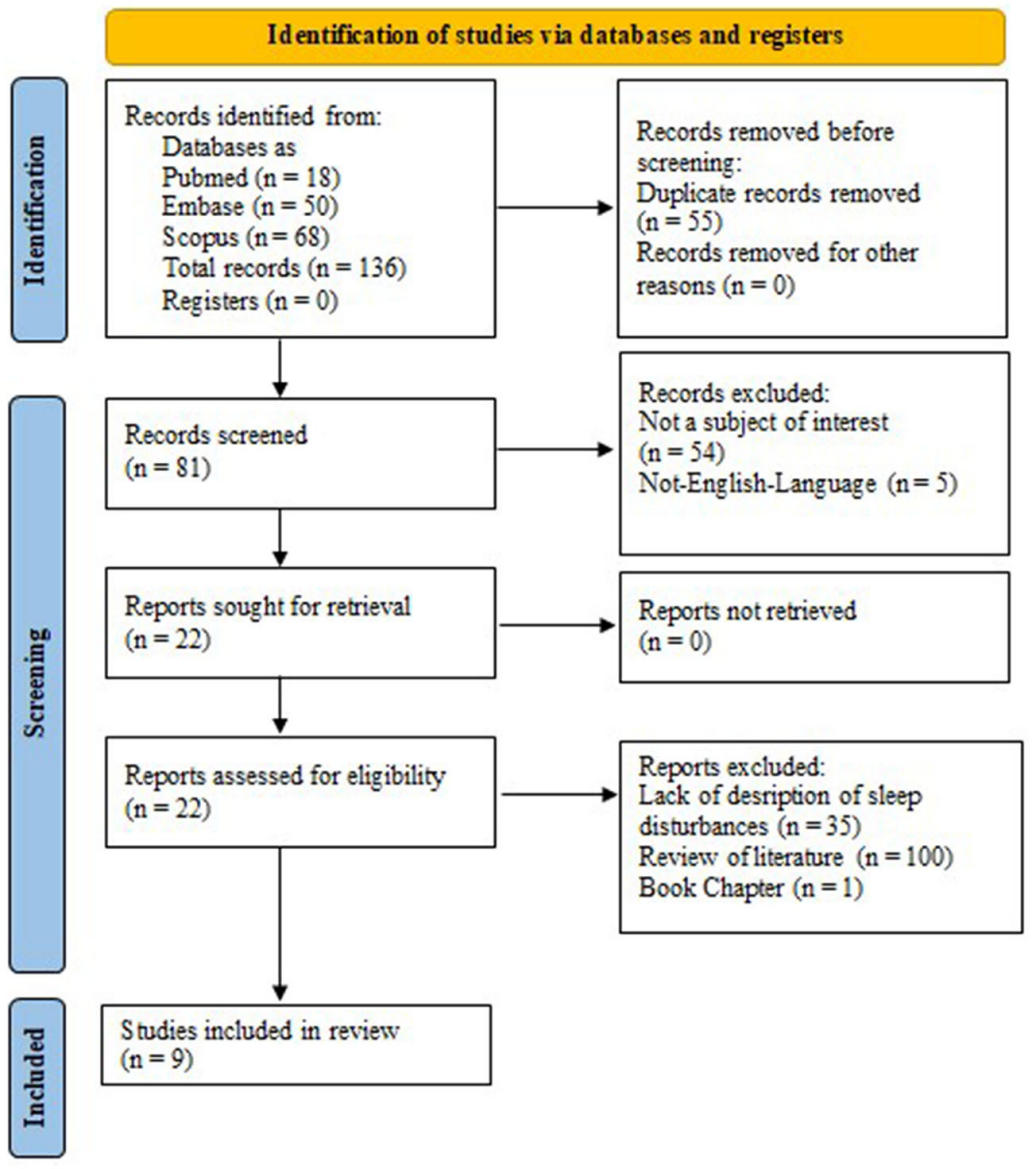
Preferred reporting items for systematic reviews and meta-analyses 2020 (PRISMA 2020) flow diagram.

After the search for key terms was carried out, a total of 136 records were identified: 18 records from PubMed, 68 from Scopus, and 50 from Embase (MEDLINE). Of these, 55 were duplicate studies, 54 articles were deemed unrelated to the purpose of our review, and five non-English studies were excluded from our analysis. The remaining 22 articles were assessed for eligibility. Of these 22 publications, eight review studies and four records did not contain a description of sleep disturbances in patients with FD, and one was a book chapter. Finally, nine studies were included in our review (16, 17, 19–24, 26). Figure 1 presents a summary of our search. The main characteristics of the included studies are outlined in Table 1.

### 3.2. Quality of studies

Of the publications included in this review, five articles were cohort studies (16, 17, 19, 20, 23), two were cross-sectional records (21, 22), one was a case–control study (18), and one was a case report (24). The evaluation of study quality was guided by the National Institute of Health (NIH) tools for cohort and cross-sectional studies. In the cohort category, three studies were assessed as fair in overall quality (17, 19, 20), while two were deemed poor (16, 23). The two cross-sectional studies were also evaluated as being of poor quality (21, 22). Further details are available in Supplementary Table 1. For the case–control study, an NIH tool specially curated for the evaluation of this type of research was used. This case–control study was ultimately (18) deemed to be of poor quality. These ratings can be seen in Supplementary Table 2. Following the JBI checklist for case reports, the case report presented herein (24) received five “yes” marks and was rated as having moderate quality. A summary of this assessment is presented in Supplementary Table 3.

### 3.3. Included studies

#### 3.3.1. Main characteristics

According to the methods described in Section 2.2, we identified nine studies detailing sleep disorders in patients with FD. These studies encompassed 330 FD patients, comprising 184 female and 146 male patients. Most studies included both male and female participants (16–18, 20, 21, 23). An exception was (19) which had solely focused on male participants and the case report (24) which had a female participant. Patients observed in the studies were of an average age of ~40 years, with appropriate standard deviations. Only the female patient in the case report was in her 50s. The studies included patients whose FD was most commonly confirmed through genetic analysis of known mutations. However, a study (23) also analyzed patients with GLA gene variants that do not lead to substrate accumulation but do present with certain disease symptoms. In some of the studies, the body mass index (BMI) of patients was given along with the standard deviation; in three of the studies (17, 19, 20), the body weight of the observed subjects was within the normal range, while in one of them (22), it was possible to conclude that the study cohort was overweight. Within the collected data, 219 out of 330 patients (66.36%) were treated with enzyme replacement therapy (ERT).

#### 3.3.2. Description and diagnostic methods of sleep disorders

Overall, sleep problems affected 213 out of 330 patients (64.5%). Excessive daytime sleepiness (EDS) was reported most frequently in six studies (17, 19–22, 24), assessed using the Epworth Sleepiness Scale (ESS). Two studies (17, 22) provided mean ESS scores, while the remaining studies (19–21, 24) mentioned the number of patients with EDS (scoring > 10 points). Hence, in these four studies, 43 out of 183 FD patients (23.5%) were diagnosed with EDS. In addition to the aforementioned scale, the multiple sleep latency test (MSLT) was also used for the diagnosis of EDS in one study (24).

Using the Pittsburgh Sleep Quality Index (PSQI) questionnaire (16, 23, 26), sleep quality over the previous month was measured and assessed through patient responses. Poor sleep quality was diagnosed in 42 out of 72 subjects (58.3%) included in these studies. The REM Sleep Behavior Disorder Screening Questionnaire (RBDSQ) was used to diagnose disturbed muscle atonia and nightmares occurring during the rapid eye movement (REM) sleep stage (21), which is common in people with neurodegenerative disorders; 29 cases of REM sleep behavior disorder (RBD) were identified among 110 participants (26.4%) (21). Medical interviews conducted in studies by Rosa Neto et al. (16, 23) identified insomnia or unrefreshing sleep, affecting 26 out of 56 subjects (46.4%).

More objective and reliable approaches, such as polysomnography (PSG) or respiratory polygraphy, were utilized to evaluate sleep apnea in specific studies. This involved examining obstructive sleep apnea (OSA) in studies (17, 19, 20, 22), central sleep apnea (CSA) with Cheyne–Stokes respiration in studies (22, 24), and CSA without Cheyne–Stokes respiration in Franzen et al. (20). Additionally, the occurrence of periodic limb movement in sleep (PLMS) was also noted in Talbot et al. (19). Across all subjects under observation, 10 cases each of obstructive sleep apnea were identified in Gaisl et al. (17) and in Franzen et al. (20), eight were documented in Talbot et al. (19), and four cases were identified in Duning et al. (22) amounting to 32 instances among 147 patients (20.4%). Moreover, central sleep apnea accompanied by Cheyne–Stokes respiration was reported in five out of 23 patients in the study conducted by Duning et al. (22, 24). Franzen et al. (20) found three cases of central sleep apnea in a group of 52 patients. In Duning et al. (22), two participants were found to simultaneously exhibit the aforementioned disorders, resulting in CSA comprising 11.8%. Talbot et al., using polysomnography, diagnosed periodic limb movement in sleep in 19 male subjects out of a total of 20 participants (95%) (19). Furthermore, actigraphy was performed, detecting sleep parameters including time in bed (TIB), total sleep time, sleep efficiency, and the awakening index in Vallim et al. (26). However, no significant differences in these parameters were observed between FD patients and the healthy population.

## 4. Discussion

Several studies in recent years have focused on the quality of life experienced by FD patients. These studies revealed that, in addition to the characteristic symptoms of the disease, sleep disturbances are also common in this group. Despite this revelation, sleep-related problems are scarcely acknowledged within the description of the disease itself. Therefore, our systematic review focused on determining the prevalence of sleep-related disorders and sought to identify the specific sleep-related conditions diagnosed among FD patients. Although the majority of the studies were assessed to be of poor or fair quality, several conclusions can still be drawn.

A majority of the reviewed research originated from five specific countries. The largest studies came from Brazil, Switzerland, and Germany (16–18, 20, 22, 23). There were also individual reports from England and Australia (19, 21). It is unclear to us as to why there are no reports on this subject from other countries, especially highly developed ones such as the USA or Japan. Arguably, the prevalence of this X-chromosome-linked recessively inherited disease varies across different countries, and the studies mentioned above originated from regions with the highest concentration of FD patients. Additionally, in these areas, sleep problems were beginning to pose a serious medical challenge. It is also interesting to note that in the reviewed studies, 184 of 330 patients were women (56%) (27, 28). This is consistent with data from Japan, where a population screening for undiagnosed cardiac, renal, and cerebrovascular diseases showed that nearly 59% of new FD diagnoses were in women (29). In contrast, Arends et al. suggested that men with the classic form of the disease experience a more severe disease trajectory compared to men with the non-classic form and women with any form of the disease (30). Therefore, it remains unclear whether the prevalence of sleep disorders is higher among women, or it appears so because men did not want to participate in conducted studies. Thus, the true prevalence of sleep disturbances is yet to be accurately estimated.

Approximately 65% of patients diagnosed with Fabry disease experienced sleep problems. In terms of age, only three of the included studies provided data on the onset of FD and the age at which sleep problems were diagnosed. Gaisl et al. (17) and Löhle et al. (21) reported that the onset of FD occurred at 15.5 ± 7.8 years and ~10–11 years before the manifestation of sleep disorders. On the other hand, Duning et al. (22, 24) indicated an onset range of 4–5 years before they conducted their studies. Therefore, these data remain inconclusive; while FD variants might provide an answer, only three articles (16, 19, 23) addressed this aspect. Unfortunately, some articles lacked a detailed diagnosis of sleep disturbances (16, 23, 26), and some of them presented inaccurate results, offering mean scores for EDS diagnosis instead of specifying the exact number of affected patients (17, 22). Notably, excessive daytime sleepiness (EDS) emerged as the most frequently reported complaint in the included studies, and it featured in six out of the nine studies. This is consistent with findings in other research studies (24), where the prevalence of this condition was estimated to be as high as 68%, whereas our calculation placed the prevalence of EDS at 23.5%. In contrast, within the European population, EDS occurs in approximately 18% of healthy individuals (20). The causes of EDS in the general population include factors such as sleep deprivation, circadian rhythm disorders, depression, nervous system abnormalities, obesity, narcolepsy, or sleep apnea (31). Notably, a study measuring body temperature and melatonin metabolite excretion revealed changes in the circadian rhythm of FD patients (26, 32). Furthermore, within this cohort, EDS was found to be more frequently associated with depression rather than with sleep-disordered breathing (especially with obstructive sleep apnea (20). While there is a well-proven association between increased EDS and a heightened risk for cardiovascular diseases and even cardiovascular mortality (33, 34), the FD studies (19, 20, 22) that encompassed sleep problems and cardiovascular patients' presentation did not establish a connection between cardiovascular system impairment and an increased prevalence of sleep problems. This implies the presence of other factors contributing to the occurrence of sleep problems, but this would require further studies. However, the included studies did not specifically indicate reported symptoms in particular patients. Even though these symptoms were described in a few articles, there were differences among the studies. Gaisl et al. (17) highlighted poor sleep quality and daytime sleepiness, Talbot et al. (19) reported symptoms consistent with restless leg syndrome, Rosa Neto et al. (23) described insomnia and unrefreshing sleep, and Löhle et al. (21) presented a range of unrelated sleep disorder symptoms such as orthostatic problems, urinary dysfunction, constipation, depression, neuropathic pain, and impaired hearing. Therefore, it is hard to define primary symptoms apart from those characteristics of FD. This diversity of symptoms may arise from genotype–phenotype variations, distinct disease processes between males and females, and other organ impairments.

Sleep-related breathing disorders (SRBD) encompass a group of conditions characterized by the occurrence of respiratory arousals, which ultimately cause disturbances in sleep architecture and sleep fragmentation (35). There are four main types of SRBD, namely, obstructive apnea, central apnea, sleep-related hypoventilation, and sleep-related hypoxemia (36). Among the reviewed studies, five out of nine reported the presence of the first two conditions, with a prevalence of 20.4% for OSA and 11.8% for CSA. OSA is a breathing disorder characterized by intermittent reduction in airway patency due to airway collapse during sleep which leads, leading to hypoxemia, arousal, and sleep fragmentation, which can cause EDS (37). Its prevalence in the general population ranges from 9 to 38% (38). The causes of OSA include abnormalities in the activation and function of upper airway dilating muscles, a large tongue, tonsillar hypertrophy, or a large neck circumference, most often associated with obesity (39). Interestingly, most FD patients in certain studies had a body mass index (BMI) within the normal range. In cases where oral anatomy was taken into account by using the Mallampati score, no significant difference between FD patients and the control group was observed (17). Furthermore, throat diameters of FD patients were found to be within the normal range (19). It is worth noting that OSA is more prevalent in men (40), while the majority of the reviewed studies included women. The prevalence of OSA increases with age in both sexes (40) with an average age of patients, upon diagnosis of OSA, being approximately 40 years (with appropriate standard deviations). However, there are no available studies on OSA in FD patients who significantly differ in age. The hypothesis that glycosphingolipid deposition in upper respiratory muscles could disrupt their function and lead to nocturnal respiratory abnormalities has also not been confirmed (20). Nevertheless, deposits do accumulate in lung lysosomes, causing smooth muscle hyperplasia in the lower end of the bronchi, resulting in obstructive lung disease (41). The statistical analysis showed no association between the involved organs in FD and the presence of OSA (19) in contrast to the normal population where cardiac and cerebrovascular diseases are associated with OSA and CSA (42, 43).

In contrast, in CSA, the cause of sleep apnea is a transient decrease or temporary cessation of the respiratory drive originating in the respiratory center of the brain (44). The prevalence of CSA varies from 5 to 10% among clinic patients (45), with 11.8% of FD patients experiencing this condition. Risk factors for CSA include male sex, a history of stroke, opioid use, or heart failure. These factors are especially linked to CSA when it coexists with Cheyne–Stokes respiration (CSR) (45). Other causes can be physiological factors, muscle, endocrine, brainstem, or spinal cord disorders (46). In the study by Duning et al., there was no association between heart failure and CSA (22). Similar conclusions were drawn by Franzen et al. (20). Because of overlapping pathophysiological causes, clinical OSA and CSA can coexist (47), as observed in FD patients in the study by Duning et al. (22).

Given the pathomechanism of FD, changes in the central nervous system could also be expected. Indeed, using “gold-standard” imaging techniques such as magnetic resonance imaging (MRI), small vessel microangiopathy-induced white matter hyperintensities (WMH) were detected, emerging as the most frequent brain lesions in this patient group (48). Additionally, reduced brain volume and the presence of the pulvinar sign were mentioned (49). However, these neurological lesions are not distinct or specific to this syndrome (50). For example, WMH can manifest in aging populations and individuals with Parkinson's disease (51, 52), and the pulvinar sign has also been noted in conditions such as Creutzfeldt–Jakob disease and antiCV2 encephalitis (53). Given these considerations, brain lesions may have a link to sleep issues. Dunning et al. (22) used a diffusion tensor imaging (DTI)-based sequence that can detect even the smallest damage to brainstem neuronal networks on MRI. They found that there is a correlation between the extent of brainstem damage in FD patients and the severity of central sleep apnea with Cheyne–Stokes respiration (CSA–CSR). Moreover, similar changes in the white matter as seen in FD have been associated with sleep disturbances in Parkinson's disease resulting in shorter sleep duration among middle-aged adults (54, 55). However, the most recent studies by Kocevska et al. (56) and Li et al. (57) demonstrated a lack of association between global white matter lesions and sleep problems. It is worth mentioning that all participants in the Duning et al. (22) study had, including non-CSA patients, white matter lesions in the brain. The studies (22, 54, 55) comprised relatively small patient groups and were published before those of Kocevska et al. and Li et al. Additionally, the specific brain regions where these lesions occur could potentially influence the outcomes related to sleep problems. Therefore, more studies assessing brain changes and sleep quality, particularly in Fabry disease, are needed.

The genotypes of the disease play an important role in treatment, and ~700 variants of FD have been identified to date. However, novel types and variants of unknown significance (VOUS) continue to be discovered (58, 59). Within the gathered group of patients, only two studies presented patient genotypes (16, 23), and one clearly showed the phenotypes (19). However, one of them (23) included variants that are not entirely associated with FD. As a result, we were unable to ascertain the relationship between specific FD variants and sleep problems or to indicate any particular trend in a certain type causing sleep problems. More studies are required to provide a comprehensive understanding of FD and its relationship with sleep problems. Thanks to genetic engineering, ERT is available for the treatment of FD. This therapy involves intravenous infusion of the missing enzyme α-galactosidase to patients. However, the amenable GLA variant could also be treated by pharmacological chaperone therapy (Migalastat), which is one of the oral regimens used for FD treatment (60). The indication for this treatment is determined by the manifestation of the disease and the accompanying symptoms of organ damage. However, in general, this therapy is recommended for all FD patients as it can reverse the organ changes caused by the disease (61). The included studies indicated that either all participants (22) or a smaller subset (23) had received such treatment. It should, however, be noted that despite receiving treatment, sleep-related symptoms such as EDS persisted (22, 24), along with other sleep disorders. Duning et al. (24) also reported a lack of correlation between years of ERT and ESS results. Moreover, no guidelines for treating sleep disorders are included in the recommendations for adjunctive treatment of disease symptoms (62). Additionally, treatment options for affected patients were not presented in the included studies. Only Gaisl et al. (17) reported the use of continuous positive airway pressure (CPAP) therapy for OSA, but without follow-up information, the results remain unknown. There are pharmacological options for treating EDS (31) as well as the “gold-standard” CPAP therapy for OSA treatment. In cases of mild OSA, mandibular advancement devices (MAD), positional therapy, and weight loss therapy are used (63). However, there is a lack of evidence in the literature regarding the effectiveness of these treatment options for this specific patient group. Therefore, there is a need for further studies to explore the response of FD patients with sleep problems to these treatment approaches.

Various methods were used for the diagnosis of sleep disorders in patients within the reviewed studies. Polysomnography (PSG) serves as the “gold-standard” technique for identifying sleep disorders (64). PSG uses various techniques to measure sleep parameters, respiratory effort and airflow, oxygen saturation, heart rate and rhythm, limb movements, body position, and comprehensive behavioral monitoring using cameras (65). Unfortunately, only three out of nine studies used this objective examination to diagnose their patients. Two studies used respiratory polygraphy instead of PSG, while one study used actigraphy. However, compared to PSG, polygraphy may be inadequate for measuring OSA as it has limitations in detecting respiratory events associated with sleep arousal (66). On the other hand, actigraphy poorly identifies awake states and periodic limb movements, making it unsuitable as a substitute for PSG (67–69). Additionally, actigraphy cannot replace the multiple sleep latency test for evaluating EDS (70). During medical interviews, data were collected using scales that assess sleep problems, such as ESS or PSQI. The PSQI is used for the assessment of sleep quality and insomnia and is an important clinical tool for diagnosing these conditions (71, 72). Similarly, the ESS is effective in detecting EDS (73). However, the use of questionnaires and medical history alone, without the use of objective supplementary tests, only allows for the detection of vaguely defined medical conditions (16, 23, 26) and deprives the patient of an accurate diagnosis and numerous treatment options. Furthermore, the use of RBDSQ in Löhle et al. (21) identified the presence of REM sleep behavior disorder in FD patients although its prevalence was not higher than in the general population. While this questionnaire is efficient for population screening, neurological conditions such as Parkinson's disease require alternative diagnostic methods (57). In Talbot et al. (19), the medical history revealed that 15 out of 20 patients reported symptoms of restless leg syndrome (RLS), while PSG indicated abnormal periodic leg movements (PLMS) in 19 out of 20 subjects. However, it is also important to note that the two conditions are not the same. PLMS occurs in 80% of RLS patients and is often present without the typical symptoms of RLS (74). In FD patients, PLMS was associated with cardiac dysfunction and was not dependent on other risk factors such as anemia, iron deficiency, or neurological diseases (75).

Unfortunately, despite conducting a comprehensive systematic review of the available literature, this study does have certain limitations. First, only a limited number of studies, encompassing just five countries, have addressed the topic of sleep disorders in FD. Moreover, the included studies had a high or moderate risk of bias. Several of these studies lacked detailed diagnoses of sleep disturbances or provided inaccurate results, such as reporting mean scores for EDS diagnosis instead of specifying the exact number of affected patients. As a result, our analysis is restricted in its scope. Given these constraints, it is recommended that further studies on this topic be carried out with a larger group of patients by using objective diagnostic techniques. Additionally, further studies will help explain the response of FD patients with sleep problems to standard treatment, timeframe between FD onset and occurring sleep diseases, and relationship between sleep problems and FD genotypes and reported symptoms related to sleep problems in particular FD patients and correlation between gender and sleep disturbances. There is also a need to develop guidelines for the treatment of sleep disorders in FD patients, given that our review shows the prevalence of these issues among these patients.

## 5. Conclusion

This review has revealed the presence of many sleep-related conditions that significantly affect the quality of life of FD patients. Despite sleep disorders being more prevalent in the general population than in patients with FD, only a few studies on this subject are available in the literature. Moreover, existing results related to this topic are limited, which makes it difficult to accurately assess its true prevalence among patients with FD. Therefore, the attention of clinicians caring for these patients should be drawn to the careful assessment of potential sleep disorders in patients diagnosed with this condition. The authors would also like to emphasize the need for further research on this topic involving a larger group of subjects, explaining reported symptoms related to sleep problems, relationship between FD onset and age when sleep disturbances occurred, ERT action on sleep problems and, above all, for the creation of guidelines for the treatment of sleep disorders in patients with FD.

## Data availability statement

The original contributions presented in the study are included in the article/Supplementary material, further inquiries can be directed to the corresponding author.

## Author contributions

BB and HM contributed to the study conceptualization and prepared the manuscript. BB, MK, HM, MW, and GL collected the data. BB, HM, and MW performed data analysis. GM supervised the study. HM and MW revised the final version of the manuscript. All authors have reviewed and approved the manuscript for publication.
