# Supplementary material for: Fabry disease and sleep disorders: a systematic review
Source: Front Neurol. 2023 Oct 5;14:1217618. doi: 10.3389/fneur.2023.1217618 (PMC10586315; doi:10.3389/fneur.2023.1217618)
Supplement: Supplementary file 1 [file Table_1.docx]

Table 1. Assessment of overall risk of bias using National Institutes of Health (NIH) quality assessment questions for Observational Cohort and Cross-Sectional Studies.

|  | | Cohort studies | | | | | Cross-sectional studies | |
| --- | --- | --- | --- | --- | --- | --- | --- | --- |
|  |  | (17) | (18) | (20) | (21) | (24) | (22) | (23) |
| National Institutes of Health (NIH) quality assessment questions for Observational Cohort and Cross-Sectional Studies | 1. Was the research question or objective in this paper clearly stated? | Yes | No | Yes | No | Yes | No | Yes |
|  | 2. Was the study population clearly specified and defined? | Yes | Yes | Yes | Yes | No | Yes | Yes |
|  | 3. Was the participation rate of eligible persons at least 50%? | Not reported | Yes | Yes | Yes | Not reported | No reported | Not reported |
|  | 4. Were all the subjects selected or recruited from the same or similar populations? Were inclusion and exclusion criteria for being in the study prespecified and applied uniformly to all participants? | No | Yes | Yes | Yes | No | Yes | No |
|  | 5. Was a sample size justification, power description, or variance and effect estimates provided? | No | Yes | No | No | No | No | No |
|  | 6. For the analyses in this paper, were the exposure(s) of interest measured prior to the outcome(s) being measured? | Yes | Yes | No | Yes | Yes | No | No |
|  | 7. Was the timeframe sufficient so that one could reasonably expect to see an association between exposure and outcome if it existed? | Not reported | Yes | Yes | Yes | No | No | Not reported |
|  | 8. For exposures that can vary in amount or level, did the study examine different levels of the exposure as related to the outcome? | Not appicable | Not appicable | Not appicable | Not appicable | Not appicable | No | Not appicable |
|  | 9. Were the exposure measures clearly defined, valid, reliable, and implemented consistently across all study participants? | No | No | No | No | No | No | No |
|  | 10. Was the exposure(s) assessed more than once over time? | No | No | No | No | No | No | No |
|  | 11. Were the outcome measures (dependent variables) clearly defined, valid, reliable, and implemented consistently across all study participants? | No | Yes | Yes | Yes | No | No | Yes |
|  | 12. Were the outcome assessors blinded to the exposure status of participants? | No | No | No | No | No | No | No |
|  | 13. Was loss to follow-up after baseline 20% or less? | Not reported | Not reported | Not reported | Not reported | Not reported | Not reported | Not reported |
|  | 14. Were key potential confounding variables measured and adjusted statistically for their impact on the relationship between exposure(s) and outcome(s)? | Yes | Yes | Yes | Yes | Yes | Yes | Yes |
| Overall quality assessment | | Poor | Fairy | Fairy | Fairy | Poor | Poor | Poor |

|  | | (26) |
| --- | --- | --- |
| National Institutes of Health (NIH) quality assessment questions l for case-control study | 1. Was the research question or objective in this paper clearly stated and appropriate? | No |
|  | 2. Was the study population clearly specified and defined? | Yes |
|  | 3. Did the authors include a sample size justification? | No |
|  | 4. Were controls selected or recruited from the same or similar population that gave rise to the cases (including the same timeframe)? | Yes |
|  | 5. Were the definitions, inclusion and exclusion criteria, algorithms or processes used to identify or select cases and controls valid, reliable, and implemented consistently across all study participants? | Yes |
|  | 6. Were the cases clearly defined and differentiated from controls? | No |
|  | 7. If less than 100 percent of eligible cases and/or controls were selected for the study, were the cases and/or controls randomly selected from those eligible? | No |
|  | 8. Was there use of concurrent controls? | No |
|  | 9. Were the investigators able to confirm that the exposure/risk occurred prior to the development of the condition or event that defined a participant as a case? | Yes |
|  | 10. Were the measures of exposure/risk clearly defined, valid, reliable, and implemented consistently (including the same time period) across all study participants? | No |
|  | 11. Were the assessors of exposure/risk blinded to the case or control status of participants | No |
|  | 12. Were key potential confounding variables measured and adjusted statistically in the analyses? If matching was used, did the investigators account for matching during study analysis? | Yes |
| Overall quality assessment | | Poor |

Table 2. Assessment of the overall risk of bias using National Institutes of Health (NIH) quality assessment questions for the single case-control study.

| ` | | (25) |
| --- | --- | --- |
| the Joanna Briggs Institute (JBI) critical appraisal tools for case report | 1.Were patient’s demographic characteristics clearly described? | Yes |
|  | 2. Was the patient’s history clearly described and presented as a timeline? | No |
|  | 3.Was the current clinical condition of the patient on presentation clearly described? | Yes |
|  | 4.Were diagnostic tests or assessment methods and the results clearly described? | Yes |
|  | 5.Was the intervention(s) or treatment procedure(s) clearly described? | Yes |
|  | 6.Was the post-intervention clinical condition clearly described? | No |
|  | 7.Were adverse events (harms) or unanticipated events identified and described? | No |
|  | 8.Does the case report provide takeaway lessons? | Yes |
| Overall quality assessment | | Moderate |

Table 3. Assessment of the overall risk of bias using the Joanna Briggs Institute (JBI) critical appraisal tools for case report.
